# The Expression of Antibiotic Resistance Methyltransferase Correlates with mRNA Stability Independently of Ribosome Stalling

**DOI:** 10.1128/AAC.01806-16

**Published:** 2016-11-21

**Authors:** Ekaterina Dzyubak, Mee-Ngan F. Yap

**Affiliations:** Edward A. Doisy Department of Biochemistry and Molecular Biology, Saint Louis University School of Medicine, St. Louis, Missouri, USA

## Abstract

Members of the Erm methyltransferase family modify 23S rRNA of the bacterial ribosome and render cross-resistance to macrolides and multiple distantly related antibiotics. Previous studies have shown that the expression of *erm* is activated when a macrolide-bound ribosome stalls the translation of the leader peptide preceding the cotranscribed *erm*. Ribosome stalling is thought to destabilize the inhibitory stem-loop mRNA structure and exposes the *erm* Shine-Dalgarno (SD) sequence for translational initiation. Paradoxically, mutations that abolish ribosome stalling are routinely found in hyper-resistant clinical isolates; however, the significance of the stalling-dead leader sequence is largely unknown. Here, we show that nonsense mutations in the Staphylococcus aureus ErmB leader peptide (ErmBL) lead to high basal and induced expression of downstream ErmB in the absence or presence of macrolide concomitantly with elevated ribosome methylation and resistance. The overexpression of ErmB is associated with the reduced turnover of the *ermBL-ermB* transcript, and the macrolide appears to mitigate mRNA cleavage at a site immediately downstream of the *ermBL* SD sequence. The stabilizing effect of antibiotics on mRNA is not limited to *ermBL-ermB*; cationic antibiotics representing a ribosome-stalling inducer and a noninducer increase the half-life of specific transcripts. These data unveil a new layer of *ermB* regulation and imply that ErmBL translation or ribosome stalling serves as a “tuner” to suppress aberrant production of ErmB because methylated ribosome may impose a fitness cost on the bacterium as a result of misregulated translation.

## INTRODUCTION

Macrolide antibiotics inhibit bacterial protein synthesis by binding to the ribosomal exit tunnel (1). The extensive use of macrolides in agribusiness and the medical community has accelerated the erosion of the efficacy of these drugs and the spread of transmissible resistant determinants (2–4). One of the major resistance mechanisms is caused by the members of the Erm methyltransferase family, which modify the single 23S rRNA nucleotide A2058 (Escherichia coli numbering) of the bacterial 50S ribosomal subunit and thereby reduce the drug-binding affinity. Dimethylation of A2058 (m^6^_2_A2058) not only evokes resistance to the prototypic macrolide erythromycin (ERY) but also leads to cross-resistance to the structurally distinct lincosamides and streptogramins, which share the overlapping binding site (5). Erm enzymes are most prevalent in Gram-positive staphylococci, streptococci, enterococci, and clostridia but are increasingly found in Gram-negative bacteria of animal and human origins (4, 6–11). Previous studies on the *ermCL-ermC* operon have shown that ErmC methyltransferase translation is activated by macrolides when the antibiotic-bound ribosome stalls at a specific site in the *ermCL* leader peptide upstream of the cotranscribed *ermC*. The arrested ribosome is thought to induce a structural rearrangement of *ermCL-ermC* mRNA and allow the ribosome access to the Shine-Dalgarno (SD) sequence that would otherwise be occluded from translational initiation (12–14). Analogous leader peptide-dependent, ribosome-stalling-mediated translational regulation has also been proposed for other homologous *erm* systems (15–19) and in other ligand-dependent (20–23) and ligand-independent (24–29) bicistrons. In other cases, ribosome stalling in the leader sequence promotes downstream transcription by precluding termination factor binding or by melting of the termination mRNA structure (23, 30, 31).

Erm-directed resistance can either be constitutive or macrolide inducible, and the ribosome-stalling-based regulation belongs to the latter category (5, 32). The distributions of constitutive and inducible resistance in natural bacterial populations are not well documented. Nevertheless, in many clinical surveillance studies, the constitutive phenotype appears to be equally widespread, if not the most predominant type, in resistant isolates from patients (33–44). Insertions, duplications, deletions, and missense mutations within the leader regulatory region are commonly found in the constitutively expressed *erm* operons (33–43). The deletion of a substantial portion of the regulatory region could explain the overproduction of Erm enzyme because the inhibitory mRNA hairpin structure is removed. However, most naturally occurring mutations are more subtle and, in many cases, result in premature termination before translation reaches the ribosome-stalling site. These leader peptide mutants, which are presumably defective in ribosome stalling, remain responsive to macrolides that further upregulate the downstream *erm* expression. The mechanism underlying these apparently paradoxical phenomena has not been established.

**FIG 1:**
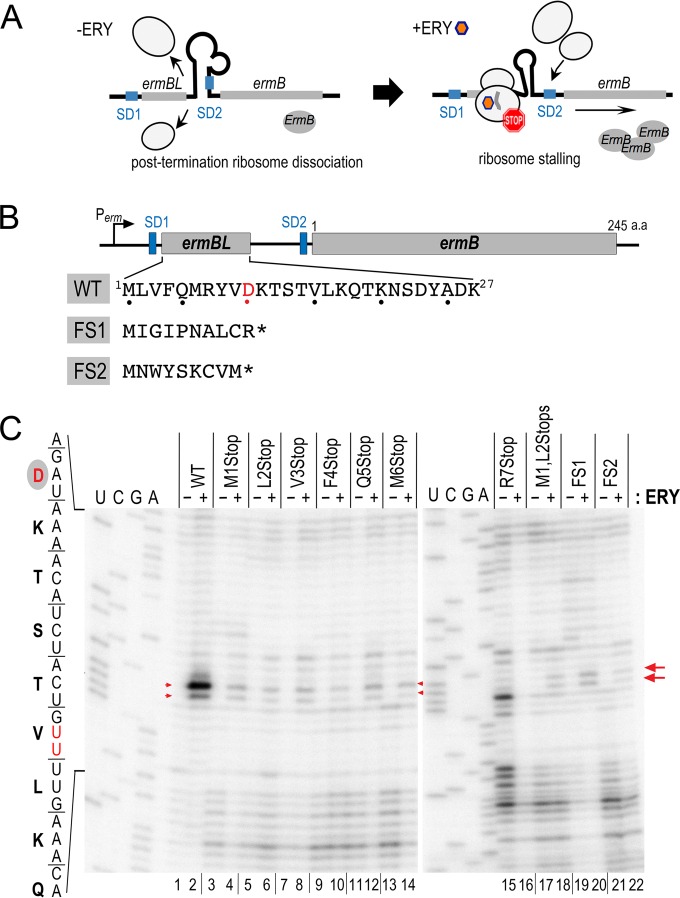
Introducing premature ochre codons in ErmBL_EF_ completely or severely impairs ribosome stalling *in vitro*. (A) A previously proposed model of ErmB upregulation by the erythromycin (ERY)-induced ErmBL ribosome stalling. ERY-bound ribosome stalls at the C terminus of ErmBL, causes structural rearrangement of the mRNA, and in turn exposes the Shine-Dalgarno (SD) sequence of downstream *ermB* for translational initiation. (B) Schematic diagram of the *ermBL_EF_-ermB* operon from S. aureus CM05. Protein sequences of wild-type (WT) ErmBL_EF_ and the corresponding frameshift (FS) mutants are shown. The ribosome-stalling site D10 is highlighted in red. SD sequences are marked. An asterisk depicts a termination codon. (B) Mapping of the ERY-stalled ribosome on its mRNA template by toeprinting. Reverse transcriptase halts at a site 16 to 17 nt (red arrows) downstream of the P-site codon of a stalled ribosome, producing a truncated cDNA product that was analyzed using a denaturing sequencing gel. Weak background bands in the nonsense mutants are partially due to translational readthrough (see Fig. S3A in the supplemental material) and are partially due to reverse transcription pausing on the naked mRNA without added ribosome (see Fig. S3B in the supplemental material). The reason for the unusual primer extension signals in the R7Stop in the absence of ERY is unclear. ERY was used at 50 μM.

We used the *ermBL-ermB* operon from methicillin-resistant Staphylococcus aureus CM05 as a model to investigate the inducible and constitutive resistance. The intergenic region of other homologous *ermBL-ermB* transcript is known to adopt secondary structure, which could mask the SD sequence of *ermB* (18) (Fig. 1A). Consistent with relevant clinical studies, we found that cells bearing truncated ErmBL (and consequently impaired in ribosome stalling) remain highly resistant via a previously underappreciated mechanism by which the antibiotic promotes the stability of *ermB* mRNA. We found that *ermBL-ermB* mRNA is more susceptible to RNase in the absence of ERY and that the nucleolytic site lies immediately downstream of the *ermBL* SD at a region coinciding with the reported consensus sequence of some RNase E substrates (45–47). Finally, we found that other cationic ribosome inhibitors, regardless of their ability to elicit ribosome stalling, can increase the mRNA abundance of a specific group of genes, most likely by enhancing mRNA stability. Our results reveal an alternative “off-target” resistance-inducing pathway and support the emerging idea that empirical antibiotic therapy can lead to unintended consequences by promoting mRNA stability, some of these mRNA coding products may be virulence factors.

## MATERIALS AND METHODS

### Bacterial strains, plasmids, growth conditions, and reagents.

E. coli NM580 is a derivative of K-12 MG1655, which carries a mini-λRed recombinase and the counterselective markers comprising a kanamycin cassette and a *ccdB* toxin gene under the control of an arabinose-inducible promoter (a gift from N. Majdalani) (see Table S1 in the supplemental material) (48). Linear DNA templates bearing the P_erm_-*ermBL-ermB*′ or P_erm_-*ermBL*′ regions were PCR amplified using p494 and p495 as primers (see Table S2 in the supplemental material) and p381ermBL-B′ as the template. A 40-nucleotide (nt) homologous sequence flanking the *kan*-pBAD-*ccdB* cassette was incorporated into the 0.3- to 0.5-kb PCR products. After PCR purification, the DNA was recombined into the chromosomal *lacZ* locus of NM580 by inducing λRed recombinase using a temperature shift to 42°C. Successful recombinations were indicated by the loss of kanamycin resistance, and the identity of the recombinants was verified by DNA sequencing.

To construct p381ermBL-B′ and pTAermBL-ermB plasmids (see Table S1 in the supplemental material), the *ermBL-ermB*′ or *ermBL-ermB* region, including its native promoter, was amplified using p129+p151 or p805+p806 as a primer pair and the genomic DNA of S. aureus CM05 as the template. The *ermBL-ermB*′ fragment was cloned into the HindIII and BamHI sites of pRB381 (49) to create a *lacZ* translational fusion at codon 13 of *ermB*. The full-length *ermB-ermBL* fragment was cloned into pGEMT-Easy via TA cloning (Promega). Site-specific mutations in *ermBL* or *ermB* were introduced by QuikChange mutagenesis (Agilent). S. aureus cells were grown in tryptic soy broth (Difco), whereas E. coli strains were grown in Luria-Bertani (LB) broth (Fisher) at 37°C unless otherwise noted. DNA primers were obtained from IDT DNA. Antibiotics and chemicals were purchased from Sigma. Antibiotics were used at the following concentrations unless otherwise stated: ampicillin (100 μg/ml), kanamycin (50 μg/ml), erythromycin (10 to 200 μg/ml), clindamycin (100 to 1,500 μM), tylosin (0.25 to 3.5 mM), and rifampin (200 μg/ml). Serial titrations of antibiotics were performed to determine the optimal drug concentration for *ermB-lacZ* induction and toeprinting. The MIC of erythromycin was determined by Etest on the Mueller-Hinton agar (BD Difco) plates according to the manufacturer's instructions (bioMérieux).

### *In vitro* translation and toeprinting analyses.

Linear DNA templates carrying the T7 promoter were programmed in 5 μl of cell-free PURExpress translation system (New England BioLabs) in the presence or absence of antibiotics. To detect translation products, the reactions were supplemented with 10 μCi of Tran^35^S-label (MP Biomedicals), incubated at 37°C for 1 h, precipitated with 4× volumes of acetone, resolved on 12% Bis-Tris SDS-PAGE gels, and autoradiographed (see Fig. S3A in the supplemental material). In the toeprinting experiments (50), the same translation reactions were assembled, except that the Tran^35^S-label was omitted. A PURExpress Δ ribosome translation mixture (NEB) without ribosome was programmed with appropriate DNA template and served as a toeprinting negative control. After incubation at 37°C for 15 min, primer extension was carried out at 37°C for 1 h using a ^32^P-labeled oligonucleotide that complemented a region 30 to 100 nt downstream of the ribosome-stalling site. The resulting cDNA was extracted once with phenol-chloroform (pH 6.8; Amresco) and was finally precipitated using 0.3 M sodium acetate (pH 5.2) and 3× volumes of isopropanol. The DNA pellet was washed with 70% ethanol, and the air-dried pellet was resuspended in 30 μl of formamide-containing loading buffer. DNA sequencing ladders were generated using a USB Thermo SEQ kit (Affymetrix). Five microliters of the toeprinting products and 1-μl portions of the ladders were heat denatured and resolved on 6% TBE (Tris-borate-EDTA)-urea polyacrylamide (19:1) sequencing gels and then scanned on a GE Typhoon phosphorimager. The intensity of m^6^_2_A2058 signal was quantitated by ImageJ.

### Northern blotting.

Oligonucleotides complementary to tRNA^Asp^, tRNA^Pro^, and tRNA^Lys^ (see Table S2 in the supplemental material) were biotinylated using a BrightStar psoralen-biotin kit (Ambion) under UV irradiation (365 nm) on ice for 45 min. The labeling efficiency was estimated by spotting serial diluted probes alongside the positive control provided in the kit, followed by signal detection with a BrightStar detection kit (Ambion). *In vitro* translation of the T7 -driven *ermBL* was carried out at 37°C for 1 h using a PURExpress kit (NEB). The translated products were resolved using neutral-pH 12% Bis-Tris SDS-PAGE (Invitrogen), and the tRNA-containing species were detected by chemiluminescence-based biotinylated oligonucleotide probe. Briefly, the translated products were transferred electrophoretically to a BrightStar-Plus nylon membrane (Ambion) using an Owl semidry HEP-1 transfer apparatus (Thermo Fisher) according to the manufacturer's protocol. Electrophoretic transfer was performed in 1× TBE buffer at 150 mA for 45 min, following UV cross-linking of the membrane inside a Stratalinker (Stratagene). Hybridization was performed at 45°C with biotinylated probes (1 pM final concentration), and washing steps were carried out using a Northern Max kit (Ambion). Finally, the hybridization signals were detected using a streptavidin-alkaline phosphatase-based BrightStar detection kit (Ambion) and by exposing the membrane to an autoradiography film (ISC BioExpress).

### β-Galactosidase assay.

E. coli strains were grown in LB until reaching an optical density at 600 nm (OD_600_) of ≈0.3. Cultures were split into two portions; one portion was treated with antibiotics (erythromycin, 100 μg/ml; clindamycin, 230 μg/ml; tylosin, 800 μg/ml), and the second portion was supplemented with an equal volume of drug solvent. After treatment at 37°C for 30 min, 0.5-ml cell cultures were harvested in triplicate, and the β-galactosidase activity was measured in these samples according to standard protocols. Miller units were calculated by normalization to the cell density (OD_600_) (51). At least three independent biological replicates were performed.

### Western blotting.

To determine the level of ErmB synthesis induction, E. coli XL1-Blue cells carrying the pTA derivatives (see Table S1 in the supplemental material) were grown and treated with antibiotics as described above. Portions (3 ml) of each culture were collected and resuspended in 1.5 ml of 20 mM Tris (pH 7.0). The suspensions were then divided in half. One portion was subjected to RNA isolation (see below), and the other portion was sonicated to obtain a crude cell lysate. Total soluble proteins (40 μg/lane) were resolved using 4 to 20% TGX SDS-PAGE (Bio-Rad), and the proteins were transferred to a nitrocellulose membrane using a Trans-Blot Turbo system (Bio-Rad). A 1/1,000 dilution of anti-ErmB (kindly provided by J. Rood) (52) and a 1/10,000 dilution of anti-RNAP_α_ (NeoClone) were used for immunoblotting. The intensity of immunoblot bands was quantitated by using ImageJ.

### RNA isolation and primer extension.

Total RNA was extracted using the hot phenol-SDS method (53) and an RNeasy kit (Qiagen). DNA contaminants were removed using two successive digestions with Turbo DNase I (Ambion), and RNA integrity was verified by nondenaturing agarose gel electrophoresis and ethidium bromide staining (54). Intact RNA was judged by determining the relative intensities of 23S and 16S rRNA bands, with a minimum accepted ratio of 1:1. A total of 250 ng of RNA input was used for primer extension (55); primer p1019 was used to detect m^6^_2_A2058. To detect mRNA degradation intermediates, a final 6- to 8-ng/μl portion of total RNA and primers p700 or p299 was used for primer extension after normalization of the *ermB* mRNA between ERY-treated and untreated samples by quantitative reverse transcription-PCR (qRT-PCR; see Table S2 in the supplemental material) (55).

### Rifampin chase and qRT-PCR.

E. coli NM580 derivatives were treated with antibiotics or mock solvent for 10 min. A final concentration of rifampin at 200 μg/ml or an equal volume of dimethyl sulfoxide was added to each culture. At time point zero (*t*_0_) and at subsequent time points (60, 90, 120, 150, 180, and 300 s), 1-ml samples of cells were subjected to hot-phenol-SDS RNA extraction (53) and RNeasy kit (Qiagen) purification. RT was performed using 5× iScript Supermix (Bio-Rad) and a 10-ng/μl concentration of DNase I-treated RNA. A “minus-RT” control was performed in parallel to ensure that the RNA was DNA-free. Quantitative PCR was performed in triplicate in 20-μl reaction mixtures containing 1× iTaq Universal SYBR green supermix (Bio-Rad), 0.4 μM concentrations of primers (see Table S2 in the supplemental material), and 1 μl of cDNA on a CFX96 real-time PCR instrument (Bio-Rad). Gene-specific primers were used (see Table S2 in the supplemental material), and 16S rRNA was used as an internal reference gene. Differences in mRNA levels were calculated using a published 2^−ΔΔ*CT*^ formula (56). mRNA half-lives were determined by fitting the data points to the equation *y* = *a*·*e* ^ (*b*·*x*), where *y* is the mRNA fraction, *x* is the time (in seconds), *a* is the initial number of mRNA, *b* is the decay constant, and *e*^ indicates exponential function; the fitted curve was used to calculate the half-life according to the equation *t*_1/2_ = *x*(1) − *x*(0.5).

### RNA-Seq analysis.

Total RNA samples from three independent biological replicates were isolated as described above. RNA integrity after DNase I treatment was confirmed using a Bioanalyzer RNA 6000 Nano kit (Agilent). Four micrograms of RNA from each sample were subjected to rRNA depletion using a Ribo-Zero kit (Illumina) according to the manufacturer's protocol. RNA-Seq (transcriptome sequencing) was conducted at the Saint Louis University Biochemistry Genomics Core. Sequencing libraries were constructed using an Ion Total RNA-Seq kit (v2; Thermo Fisher) and were sequenced using an Ion Torrent Proton instrument (Life Technologies) with a mean read length of 101 bp and a minimum of 114× coverage. Alignment to the E. coli MG1655 reference genome (GenBank NC_000913.3) was performed using the TMAP aligner map4 algorithm (Life Technologies). Although Ion Torrent sequencing generates reads of different lengths, conventional RNA-Seq analyses typically use total reads (reads per kilobase per million/fragments per kilobase per million) to calculate expression levels under the assumption that all reads are the same length. To accurately measure expression levels, custom R scripts were used to calculate the total nucleotide coverage for each gene. The coverage values for all genes, expression ratios, standard errors, and *P* values are presented in Dataset S1 in the supplemental material. Data analyses are described in greater detail in reference 57. Functional groups of the differentially expressed genes with *P* ≤ 0.05 and 2-fold changes were classified by Panther GOC enrichment analysis.

### Accession number(s).

Sequencing data were deposited in the NCBI database under accession number GSE80251.

## RESULTS

### Abrogating ErmBL-mediated ribosome stalling does not reduce ErmB expression.

The 27-amino-acid ErmBL leader peptide from S. aureus CM05 (GenBank accession number EF450709, referred to here as ErmBL_EF_) is highly conserved with Gram-positive bacterial homologs (Fig. 1B; see also Fig. S1 in the supplemental material) and only differs from the well-studied ErmBL at position 8 (Y8 instead of N8) (58, 59). An *in vitro* toeprinting assay was used to map the position of the stalled ribosome on *ermBL_EF_* mRNA in the presence of ERY. Consistent with previous *in vitro* results (58, 59), ERY arrested the ribosome with the D10 codon situated at the P-site (Fig. 1C; see also Fig. S2A in the supplemental material) and with a terminal aspartyl-tRNA^Asp^ attached to the ErmBL_EF_ nascent chain (see Fig. S2B in the supplemental material). Alanine mutagenesis showed that only residues R7, V9, D10, and K11 are critical for complete translation arrest (see Fig. S2A in the supplemental material). None of these alanine substitutions has been reported in the natural isolates; however, nonsense mutations, insertions, and deletions of ErmBL have been frequently found in clinical strains that exhibit high levels of multidrug resistance (34, 35, 37, 39, 40, 43, 60). Constitutive resistance derived from a complete loss of the *ermBL* regulatory region has been interpreted as a permanent disruption of the inhibitory mRNA structure that leads to downstream ErmB activation. The reasons underlying the hyper-resistance of less dramatic mutations, such as the introduction of a premature stop codon preceding the D10 ribosome-stalling sites, are poorly understood.

To investigate the consequences of introducing nonsense mutations and eliminating ErmBL translation, we replaced the first seven codons of ErmBL_EF_, one at a time, with an ochre codon that mimics the naturally occurring mutations (34, 37, 60). In principle, none of these mutants permits the ribosome to reach the ErmBL_EF_ D10 stalling site. In the frameshift mutants FS1 and FS2, one or two adenine nucleotides were inserted immediately after the AUG initiation codon, which not only alters the sequence identity but also terminates the translation at positions 11 and 10, respectively (Fig. 1B). The M1L2Stop double mutant comprises an amber and an ochre codon, and V3Stop and Q5Stop are spontaneous *ermBL* mutations that are found in clinical isolates (34). *In vitro* toeprinting (Fig. 1C) demonstrated that the D10 toeprint signals were completely (R7Stop, lanes 15 and 16) or significantly diminished in all nonsense and frameshift mutants. The latter observation did not fully meet our initial expectation that the D10 toeprints would disappear completely in all early terminated *ermBL*_EF_ mutants (Fig. 1C). Our subsequent investigations revealed that the residual background signals were derived in part from translational read-through past the stop codon (see Fig. S3A in the supplemental material) (61), and in some cases, resulted from the impeded reverse transcription on different mutated mRNA templates because toeprinting reactions that are programmed without a ribosome also produce weak signals (see Fig. S3B in the supplemental material).

**FIG 2:**
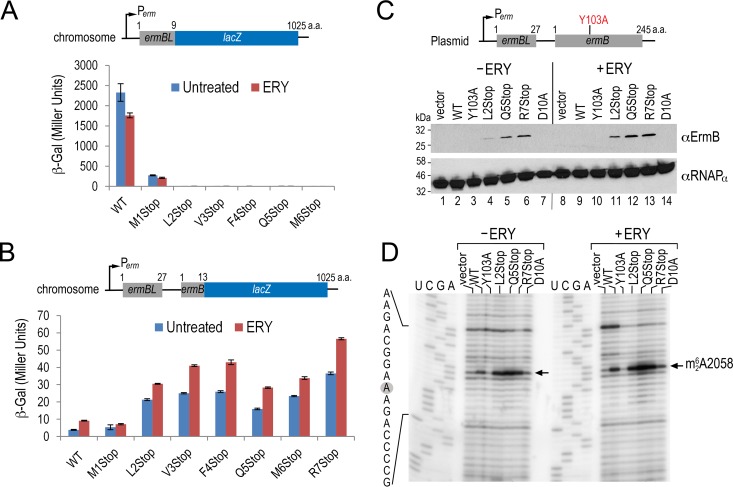
ErmBL_EF_ nonsense mutations result in a high basal and inducible expression of downstream ErmB that is consistent with the cellular concentrations of ErmB and the degree of ribosome methylation. (A) A β-galactosidase activity assay showing that nonsense mutations at residues L2 through R7 effectively shut down *lacZ* synthesis and revealing that L2 acts as an alternate start codon. β-Galactosidase activity (in Miller units) was conducted with E. coli bearing the chromosomal *ermBL′-lacZ* with or without 30 min of erythromycin (ERY) exposure. ERY was used at 100 μg/ml. (B) Results from a β-galactosidase assay showing that none of the premature nonsense mutations after codon M1 abolishes the downstream *ermB′-lacZ* expression. The β-galactosidase activity was determined as described in panel A except that chromosomal *ermBL-ermB′-lacZ* fusion was used. Error bars indicate standard deviations from three replicates. (C) Western blot analysis shows that ErmB overexpression remains inducible in response to ERY and that the degree of induction correlates with the *lacZ* reporter results. Log-phase cells with or without 30 min of ERY treatment were harvested and sonicated, and 40-μg portions of total soluble proteins were loaded per lane on the SDS-PAGE gel. Y103A is a catalytically inactive ErmB mutant. The alpha-subunit of RNA polymerase served as the loading control. A 1/1,000 dilution of anti-ErmB and a 1 1/10,000 dilution of anti-RNAP_α_ were used for immunoblotting. (D) Results from a primer extension assay showing that the magnitude of A2058 methylation is consistent with the cellular levels of ErmB. Total RNAs were isolated from the same cells shown in panel C and used at 250 ng per lane. In primer extension, the reverse transcriptase halts at the methylated site and produces a truncated cDNA that is manifested by a strong signal at A2058.

To reassure ourselves that the mutations were defective in full-length translation of ErmBL_EF_, we examined the effects of *ermBL_EF_* nonsense mutations *in vivo*. Multidrug-resistant S. aureus CM05 carries Cfr RNA methyltransferase and the macrolide efflux pump proteins MefA and MsrA, which might mask the effect of *ermBL_EF_-ermB*. To avoid complications in interpreting the resistance phenotype, we either expressed the *ermBL_EF_-ermB* on a plasmid under the control of its native promoter or recombined the *lacZ* reporter alleles (*ermBL_EF_-ermB′-lacZ* or *ermBL_EF_′-lacZ*) to the native chromosomal *lacZ* locus of an E. coli surrogate host (Fig. 2). A similar approach is widely used to study antibiotic-induced resistance because E. coli cells and ribosomes are known to faithfully recapitulate peptide-dependent ribosome stalling *in vivo* and *in vitro* (14, 50, 58, 59, 62). Moreover, *ermBL-ermB* has been routinely found in many environmental and hospital E. coli strains (4, 6–10, 63, 64). By creating a translational fusion of the first nine codons of *ermBL_EF_* (excluding the D10 stalling site) directly to the *lacZ*, we confirmed that the nonsense mutations indeed abolish the translation of *lacZ* (as shown by a lack of β-galactosidase activity) with the exception of the M1Stop mutant. The mutant retains about 10% of the original β-galactosidase activity, presumably because L2 (UUG, 3% usage frequency in E. coli) acts as an alternate start codon (Fig. 2A). Furthermore, using the *lacZ* reporter fused to *ermB*, we found that the wild-type (WT) *ermBL_EF_* and M1Stop mutant moderately induced downstream *ermB′-lacZ* expression upon ERY exposure. In contrast, all other nonsense mutants demonstrated high basal expression of *ermB′-lacZ* without ERY treatment, and the levels were further elevated in the presence of ERY. Overall, ERY treatment increases *ermB′-lacZ* expression by ∼2-fold in the WT *ermBL_EF_* context and by ∼30% in the nonsense mutants (Fig. 2B), an induction level that is comparable to that of other *erm* systems (13, 14). These results indicate that the active translation of *ermBL_EF_* (WT and M1Stop) attenuates the capacity of ErmB synthesis, albeit ERY treatment can partially alleviate the repression.

To eliminate the possibility of artifacts associated with reporter mRNA and protein turnovers, we examined the expression of ErmB methyltransferase on a plasmid-borne *ermBL_EF_-ermB* in the E. coli background. An antibody against C. perfringens ErmB (52) was used to probe the expression level of ErmB. A catalytically inactive mutant of ErmB (Y103A, Y104 numbering in ErmC [65]) served as a control. Consistent with the β-galactosidase results, the basal levels of ErmB in the nonsense mutants were much greater than those in the ErmB (Y103A) mutant and WT in the absence of ERY (Fig. 2C, compare lanes 2 to 3 to lanes 4 to 6). The detection of ErmB in the WT and Y103A backgrounds was hampered by their low basal expression and in part by the low antibody titer. Nevertheless, we observed an ∼2-fold increase in ErmB induction in the nonsense mutants after 30 min of ERY exposure (compare lanes 4 to 6 to lanes 11 to 13). The expression level of ErmB was also consistent with the degree of *in vivo* ribosome methylation (Fig. 2D). Dimethylation of A2058 in 23S rRNA halts reverse transcription and produces a strong termination pause at this residue. We found that ERY treatment induces an ∼2-fold increase in methylation in the nonsense mutants and the WT, whereas the strains harboring the catalytically dead Y103A mutant and the empty vector did not undergo methylation.

**FIG 3:**
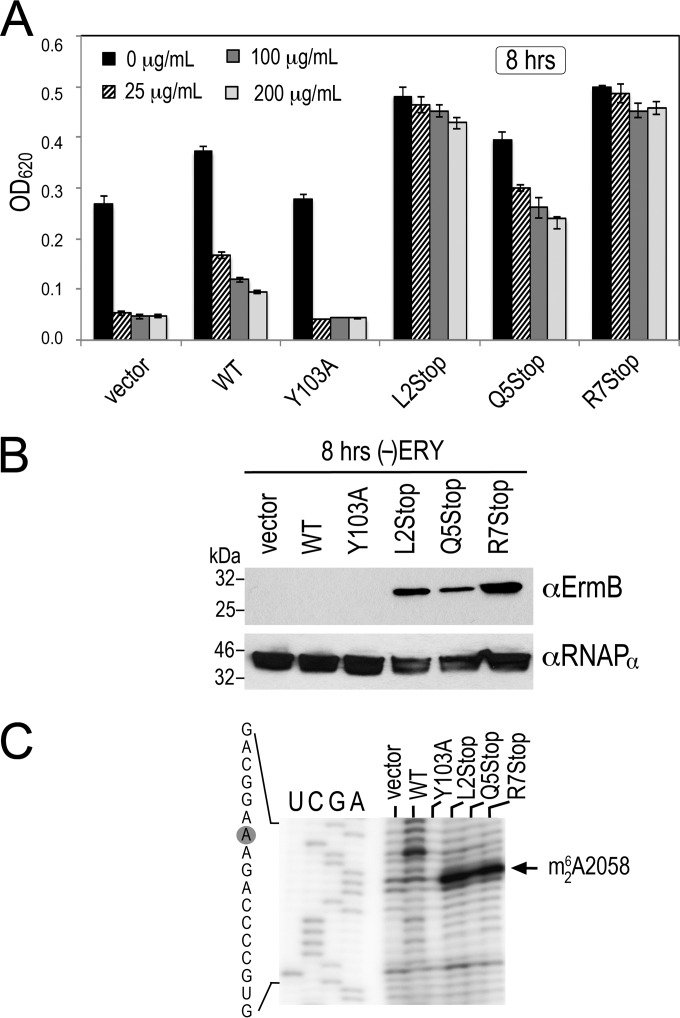
ErmBL_EF_ nonsense mutations convert the susceptible WT into ERY-resistant cells. (A) Bacterial growth in the presence of various concentrations of erythromycin (ERY). Overnight E. coli LB cultures were diluted (1/100, normalized to an OD_600_ of 0.002) into fresh medium supplemented with ampicillin at 100 μg/ml to maintain the *ermBL-ermB* bearing plasmid. The cell density was recorded at 4, 8, and 24 h after inoculation. Only the 8-h dataset is shown. Error bars indicate standard deviations obtained from three independent experiments. (B) Detection of basal ErmB levels by immunoblotting after 8 h of growth in the absence of erythromycin (ERY). Total soluble proteins were extracted from the same plasmid-borne *ermBL-ermB* backgrounds shown in panel A. Each lane corresponds to 40 μg of total soluble proteins. Y103A is a catalytically inactive ErmB mutant. The alpha-subunit of RNA polymerase served as the loading control. A 1/1,000 dilution of anti-ErmB and a 1 1/10,000 dilution of anti-RNAP_α_ were used for immunoblotting. (C) Results from a primer extension analysis showing the basal methylation of ribosomes (without ERY) after 8 h of growth. Total RNA was isolated from the same strain backgrounds shown in panels A and B. Each lane corresponds to 250 ng of RNA input.

Ribosomal methylation appears to direct the hyper-resistance phenotypes of the ErmBL_EF_ nonsense mutants. The same strains in Fig. 2C were treated with various concentrations of ERY. The MIC of ERY on the E. coli strain that we used was 46 to 64 μg/ml, as measured based on the Etest. The MIC of the *ermBL*_EF_-derived strains is >640 μg/ml. As expected, the vector and Y103A controls were extremely susceptible to subinhibitory doses of ERY, but the nonsense mutants were all resistant to high concentrations of ERY (Fig. 3A). The resistance phenotype was due to an increased level of ErmB (Fig. 3B) and an increase in ribosomal methylation (Fig. 3C). In these experiments, only untreated cells were analyzed because insufficient WT or control cells were recovered after 8 h of ERY inhibition. These data confirm that moderate 2- to 4-fold changes in ErmB expression can significantly affect bacterial resistance. Together, our results demonstrate that the ErmBL_EF_ nonsense mutants are defective in ribosome stalling, but the mutations do not reduce ErmB expression. Rather, ErmB is constitutively expressed and is moderately inducible by ERY, thereby leading to higher resistance than that observed for WT *ermBL_EF_-ermB*.

### Different ribosome-targeting antibiotics increase the steady-state level of *ermB* mRNA.

**FIG 4:**
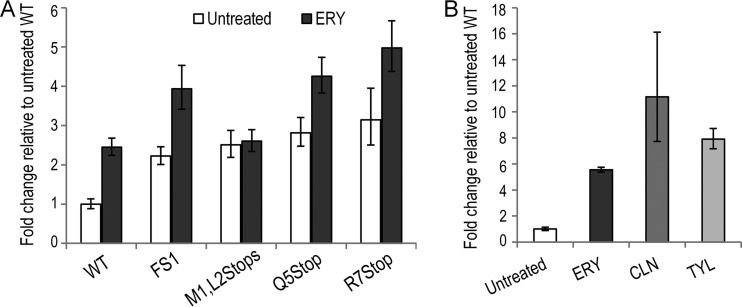
Ribosome-targeting antibiotics increase steady-state *ermB* mRNA levels independently of ErmBL_EF_-mediated ribosome stalling. (A) Quantitative RT-PCR analysis demonstrated an increase in *ermB* mRNA abundance after ERY treatment in different *ermBL*_EF_ mutant backgrounds. The fold change relative to the untreated WT is shown. (B) Quantitative RT-PCR analysis demonstrated that the noninducers of ErmBL_EF_-mediated ribosome stalling, clindamycin (CLN) and tylosin (TYL) (see Fig. S4 in the supplemental material) (58), upregulate *ermB* expression. Total RNAs were isolated from the WT *ermBL_EF_-ermB* strain treated with different antibiotics. The fold change relative to the untreated sample is shown. Error bars indicate the standard deviation of three replicates.

To understand how the ErmBL_EF_ nonsense mutations stimulate ErmB overexpression, we performed qRT-PCR to determine whether the steady-state level of *ermB* mRNA was altered in different *ermBL_EF_* mutant E. coli backgrounds treated with or without ERY. Consistent with the results described earlier (Fig. 2), approximately 2- to 3-fold increases in the mRNA levels were observed in the mutants relative to the WT in the absence of ERY. In the presence of ERY, the mRNA levels of the mutants (except for M1L2Stop) were markedly elevated by an additional 1.5-fold (Fig. 4A). Interestingly, mRNA accumulation was also observed in cells that were exposed to other noninducers of the ErmBL-mediated ribosome stalling. We observed a similar trend of antibiotic-induced mRNA upregulation in response to clindamycin (CLN) and tylosin (TYL) treatments (Fig. 4B). CLN and TYL are ribosome inhibitors and, like ERY, are positively charged. However, CLN (a lincosamide) and TYL (a 16-membered macrolide) both fail to promote ErmBL_EF_-dependent ribosome stalling (see Fig. S4 in the supplemental material) (58). These results strongly suggest that ErmB upregulation can be separated from the ribosome-stalling pathway.

### ERY promotes *ermB* mRNA stability.

**FIG 5:**
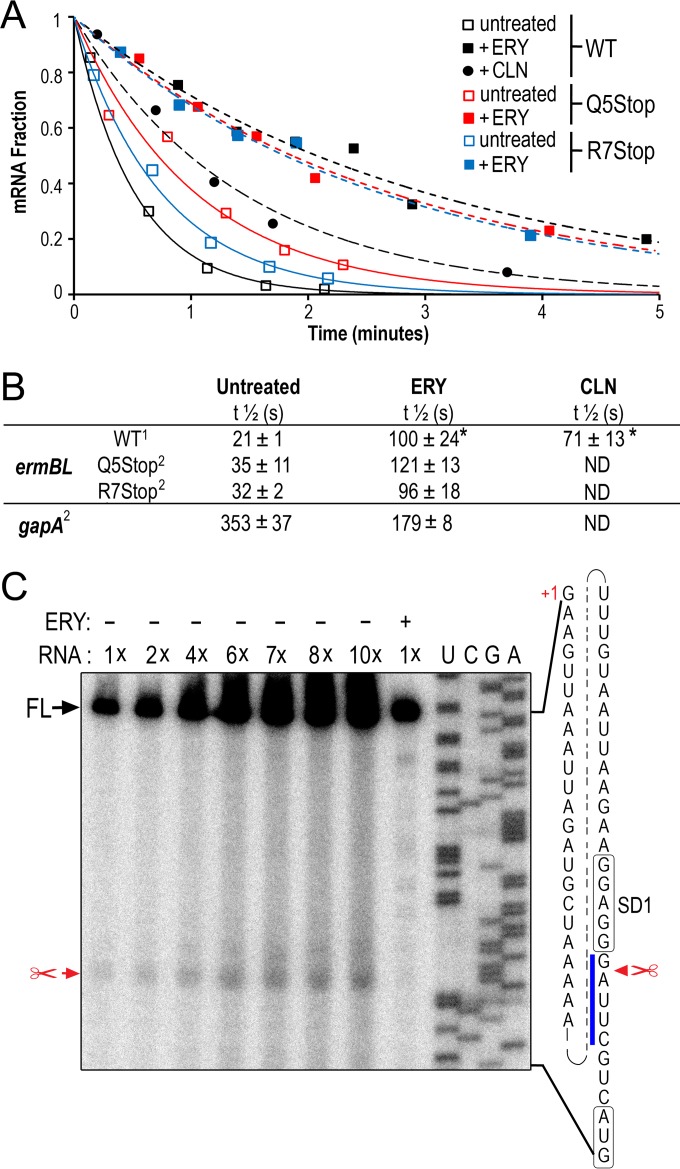
Antibiotics prolong *ermB* mRNA half-life. (A) The mRNA decay curves of ErmBL_EF_ WT, Q5Stop, and R7Stop background strains after 10 min of exposure to erythromycin (ERY) and clindamycin (CLN). A rifampin chase experiment was performed in E. coli cells bearing the chromosomally located *ermBL-ermB*′. (B) Summary of the *ermB* mRNA half-lives determined from panel A. An asterisk (*) indicates statistical significance (*t* test *P* ≤ 0.05). Superscript numbers: 1, standard deviations obtained from three independent biological replicates; 2, means and standard deviations obtained from two replicates. ND, not determined. (C) Reverse transcription mapping shows that mRNA cleavage downstream of the *ermBL_EF_* SD is reduced in the presence of erythromycin (ERY). A 1× RNA input equals 4 μg of RNA template in primer extension. The start codon and SD of *ermBL_EF_* are boxed. The RNase E-targeting sequence is denoted by a blue line. FL, full-length cDNA. The “+1” indicates the transcription start site of *ermBL_EF_-ermB*.

The observed increase in *ermB* mRNA abundance might be due to increased transcription initiation. Alternatively, ERY treatment and the ErmBL_EF_ nonsense mutations might stabilize *ermB* mRNA. To distinguish between these alternatives, we performed a rifampin chase to measure the mRNA stability over time after the transcription inhibitor rifampin was added to the cell cultures. The vast majority of E. coli mRNAs exhibit half-lives of between 3 and 9 min (66); here, we found that the level of *ermB* mRNA decreased dramatically within the first minute after rifampin treatment (Fig. 5A). After fitting the data points, a half-life of 20 ± 1 s was observed for the untreated WT *ermBL_EF_-ermB* background from three independent experiments. The half-lives were extended by about 5- and 3.5-fold, respectively, after 10 min of exposure to ERY and CLN (Fig. 5B). The half-lives of *ermB* mRNA were slightly enhanced in the untreated Q5Stop and R7Stop mutant strains relative to the WT, and the mRNA was stabilized >3-fold after ERY treatment. In contrast, *gapA*, a housekeeping gene that has been frequently used as an internal reference for qRT-PCR (67, 68), showed an opposite effect; that is, the mRNA half-life was reduced 2-fold by ERY treatment (Fig. 5B). Because mRNA half-life is linked to degradation, we performed primer extension mapping with total cellular RNA isolated from WT *ermBL_EF_-ermB* cell cultures to identify degradation intermediates of the *ermBL_EF_-ermB* transcript. The steady-state level of *ermB* mRNA in untreated cells was lower than that in ERY-treated cells (Fig. 4A); therefore, we calibrated the signals by titrating the amount of RNA inputs from the untreated cells. Consistent with previous findings (19), the transcription start site of *ermBL_EF_-ermB* was mapped at a guanine located 50 nt upstream of the AUG start codon (Fig. 5C). A processed *ermBL_EF_-ermB* mRNA intermediate was detected at a site one nucleotide downstream of the predicted core SD sequence (69). Close inspection showed that the sequences flanking the cleavage site conspicuously resembled the previously reported RNase E target sequence ([A/G]AUU[A/U/C] (45–47). RNase E cleavage sites often occur in A/U-rich single-stranded regions; however, no definitive consensus has yet been identified (66, 70). Nevertheless, the results of the primer extension experiment implied that the 5′ end of the *ermBL_EF_-ermB* transcript is a substrate of RNase E when the ERY is omitted, which may account for the shorter mRNA half-life (Fig. 5B). The data also support the notion that ribosome-targeting antibiotics can enhance mRNA stability. However, it remains unclear whether the effect is direct or indirect and whether the phenomenon can be generalized to mRNAs other than the *ermBL_EF_-ermB* transcript.

### ERY and CLN upregulate a specific subset of genes.

**FIG 6:**
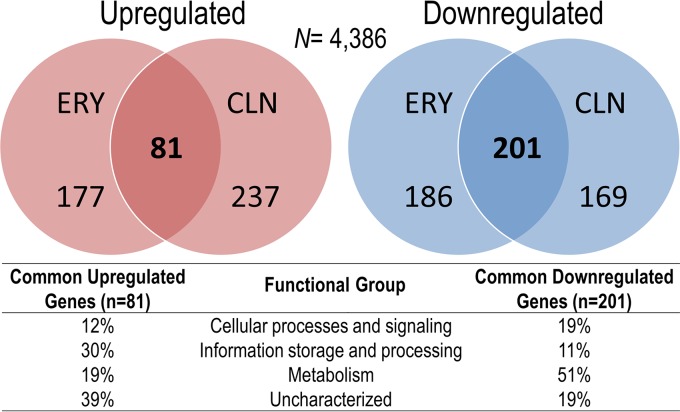
Macrolide and lincosamide differentially regulate specific gene subsets. Genome-wide transcriptome analysis shows that ERY and CLN coregulate the expression of a subset of genes that is enriched in information storage and processing and metabolic pathways. Venn diagrams show genes that exhibited a 2-fold increase or decrease in nucleotide coverage density (*P* ≤ 0.05). Complete and sorted lists of the up- and downregulated genes are provided in Dataset S1 in the supplemental material.

To determine the global effects of antibiotics, we performed an RNA-Seq analysis in E. coli (*ermBL-ermB*::*lacZ*) cells to measure changes in gene expression in response to ERY and CLN treatments. We found ca. 15% of the genes in each antibiotic treatment exhibited significant (*P* ≤ 0.05, 2-fold cutoff) increased or decreased mRNA levels (Fig. 6; see Dataset S1 in the supplemental material). Of note, a 2.3-fold increase (*P* = 0.007) in the *ermBL_EF_-ermB′-lacZ* transcript was observed in ERY-treated cells, validating our earlier results (Fig. 2 and 4A). Nearly half of the genes from each treatment were coregulated by both ERY and CLN. Of those, 30% of coupregulated genes are involved in information storage and processing, but many genes (30%) remain uncharacterized. In addition, half of the codownregulated genes are involved in cellular metabolism (Fig. 6; see Dataset S1 in the supplemental material). The expression patterns of 17 actively transcribed genes were further verified by qRT-PCR and were proven to follow the same profiles as the RNA-Seq data (see Fig. S5A in the supplemental material). Low concentrations of antibiotics can activate or repress the transcription of genes that are not their direct targets (71–76). We performed a rifampin chase to compare the mRNA decay rate in ERY-treated and untreated cells to examine whether the changes in mRNA abundance were caused by the altered stability. We found that 5 of 6 ERY-upregulated genes in ERY-treated cells exhibited significantly longer half-lives than those in untreated cells (see Fig. S5B in the supplemental material). The results suggest that the gene (*deaD*) whose mRNA half-life remained unaltered might be under transcriptional activation; however, most of the upregulated genes exhibited slowed mRNA decay when subjected to ERY treatment.

## DISCUSSION

The molecular- and atomic-level descriptions of macrolide-induced ribosome stalling have been elucidated in great detail (12–17, 19, 58, 59, 77–81); however, the relationship between ribosome stalling and the cellular levels of Erm methyltransferase (and thus bacterial resistance) has not been entirely consistent with clinical findings, wherein inducible constitutive resistance is commonly found in strains bearing ribosome-stalling-dead leader peptides. Our *in vitro* and *in vivo* analyses of the ErmBL_EF_ nonsense mutants unequivocally demonstrate that increased mRNA stability could account for the observed ErmB overproduction, that distant macrolide relatives also promote the stabilization of the *ermBL_EF_-ermB* transcript, and that antibiotic exposure exerts a protective role on mRNA decay.

The translational attenuation model of *erm* regulation has been largely inferred from the *ermCL-ermC* system, for which the mRNA structural rearrangements in the presence or absence of ERY have been mapped *in vivo* and *in vitro* (13, 82). A similar conformational switch has been detected in *ermBL-ermB* mRNA (18). However, the two operons differ from each other in at least three aspects. First, the activation of *ermC* expression is induced by a narrow spectrum of macrolides via ribosome stalling and is induced by telithromycin via a frameshifting mechanism (77, 83). In contrast, *ermB* expression is induced by macrolides, lincosamides, and streptogramins (MLS) (18, 19, 84, 85) (Fig. 4B) and by the latest generation of macrolides, termed ketolides (58, 59). In this case, only macrolides and ketolides can act as an inducer of ErmBL-dependent ribosome stalling. It is unclear how lincosamides and streptogramins upregulate *ermB* expression because the two drugs do not appear to cause mRNA conformational changes (18) or induce ErmBL-dependent ribosome stalling (see Fig. S4 in the supplemental material).

Second, the synthesis of ErmC is strictly dependent on the translation of *ermCL* because an introduction of an ochre codon at the second position of ErmCL completely abolishes inducibility and ErmC production (12). *ermBL-ermB* nonsense mutations preceding the ErmBL D10 stalling sites are found in hyper-resistant clinical isolates (34, 37, 60), and ErmB overexpression has been observed when residues that are critical for ribosomes stalling (D10 and K11) are replaced with a termination codon (18). Intriguingly, the mRNAs from D10(UAA) and K11(UAA) mutants are processed in a distinctive manner in that the level of cleaved mRNA intermediates is increased after ERY treatment in the K11(UAA)-bearing cells but not in the D10(UAA) cells. The reason for this difference is unknown, but the high basal expression of *ermB* in these mutants has been interpreted as a structural disruption of the inhibitory stem-loop when the ribosome is paused at the tenth and eleventh termination codons, which are embedded inside the predicted hairpin structure (18). The ErmBL codons M1 through M6 are located outside the stem-loop structure (18); thus, the ochre codon we introduced (Fig. 2) and the previously reported spontaneous mutations V3(UAA) and Q5(UAA) (34) are unlikely to cause drastic changes within the presumptive mRNA hairpin, although some of these nonsense mutations may alter mRNA stability. For instance, we found that in ERY-free cells, the half-lives of Q5Stop and R7Stop are slightly longer than that of WT *ermBL_EF_* (Fig. 5B). Likewise, previous *ermBL-ermB* mRNA structural probing was conducted in the B. subtilis host and in the WT *ermBL-ermB* context (18); it is possible that *ermB* SD2 is unmasked in the nonsense mutants, which could explain the high basal expression of ErmB in the absence of ERY inducer. Nevertheless, we found that the early translational termination of ErmBL_EF_ results in constitutive resistance and correlates with ErmB production and the extent of ribosome methylation. These data strongly suggest that ribosome stalling is not the sole determinant of the inducible resistance; the slowing down of mRNA turnover might represent an alternative pathway of conferring bacterial resistance.

Third, the improvement of mRNA stability upon ERY treatment has been previously reported for *ermCL-ermC* (86) and *ermBL-ermB* (18). Active *ermCL* translation is required for the observed stabilization, and stalling of the ribosome has been proposed to physically protect the mRNA from RNase action. The same interpretation has been posited for other *ermBL-ermB* system (18). In contrast, our data show that increased mRNA stability is not due to the translation of *ermBL_EF_* and ribosome stalling. Of note, the eighth position in the ErmBL_EF_ that we used is a tyrosine rather than an asparagine (as used in reference 18; see Fig. S1 in the supplemental material). In addition, under our experimental settings, we did not detect the same processed intermediates possibly due to differences in the mRNA degradation enzymes between B. subtilis and E. coli (87). More strikingly, Min et al. (18) only observed mRNA intermediates in cells that had been treated with ERY; in that study, the processing sites were mapped at the edges of the ERY-stalled ribosome. In contrast, we detected an intermediate that might be a substrate of E. coli RNase E in cells without ERY treatment, and this intermediate is absent in ERY-treated cells (Fig. 5C). The lack of an mRNA decay product in the ERY-treated cells provides an explanation for the observed increase in the steady-state mRNA level. The mechanism by which ERY reduces mRNA degradation has yet to be determined. It is possible that ERY prevents mRNA decay by altering the mRNA conformation through direct RNA binding. Although macrolide binding to other structured RNAs has not been reported, the direct binding of aminoglycosides to other viral RNAs, ribozymes, and synthetic riboswitches has been described (88–92). Alternatively, ERY might indirectly stabilize mRNA by influencing the expression of protein factors and regulatory sRNAs that are responsible for the activity and expression of RNase. Finally, the possibility that ERY may directly inhibit nucleolytic activity or inhibit the binding of RNase to the mRNA cannot be ruled out.

The nonsense ErmBL_EF_ mutations increase A2058 dimethylation, but the modification is relatively low in the WT ErmBL_EF_ and in M1Stop, which remain active in terms of translation and ribosome stalling (Fig. 2A and B). Based on our results, translation of *ermBL_EF_* and ribosome stalling appear to suppress ErmB expression, whereas disrupting these functions results in “uncontrollable” ErmB expression. The ribosome-stalling mechanism thus may be a negative regulator to ensure that only an appropriate amount of ErmB is synthesized because methylated ribosome is known to compromise bacterial fitness by perturbing translational activity (93). Furthermore, our observation that ERY and CLN both have the ability to preferentially upregulate gene expression (Fig. 6; see Dataset S1 in the supplemental material) highlights an unexpected role of antibiotics in linking mRNA metabolism to resistance and underscores a need to examine the pleiotropic effects of antibiotic therapy.

## Supplementary Material

Supplemental material
