# Supplementary material for: The Expression of Antibiotic Resistance Methyltransferase Correlates with mRNA Stability Independently of Ribosome Stalling
Source: Antimicrob Agents Chemother. 2016 Nov 21;60(12):7178–88. doi: 10.1128/AAC.01806-16 (PMC5118997; doi:10.1128/AAC.01806-16)
Supplement: Supplemental material [file supp_60_12_7178__index.html]

Supplemental material 

# The Expression of Antibiotic Resistance Methyltransferase Correlates with mRNA Stability Independently of Ribosome Stalling

## Supplemental material

- Supplemental file 1 -

  Fig. S1-S5 and Tables S1 and S2

  PDF, 13M
- Supplemental file 2 -

  Data set S1

  XLSX, 3.6M
