# Supplementary material for: The Expression of Antibiotic Resistance Methyltransferase Correlates with mRNA Stability Independently of Ribosome Stalling
Source: Antimicrob Agents Chemother. 2016 Nov 21;60(12):7178–88. doi: 10.1128/AAC.01806-16 (PMC5118997; doi:10.1128/AAC.01806-16)
Supplement: Supplemental material [file AAC.01806-16_zac012165732so1.pdf]

SUPPLEMENTAL FIGURES

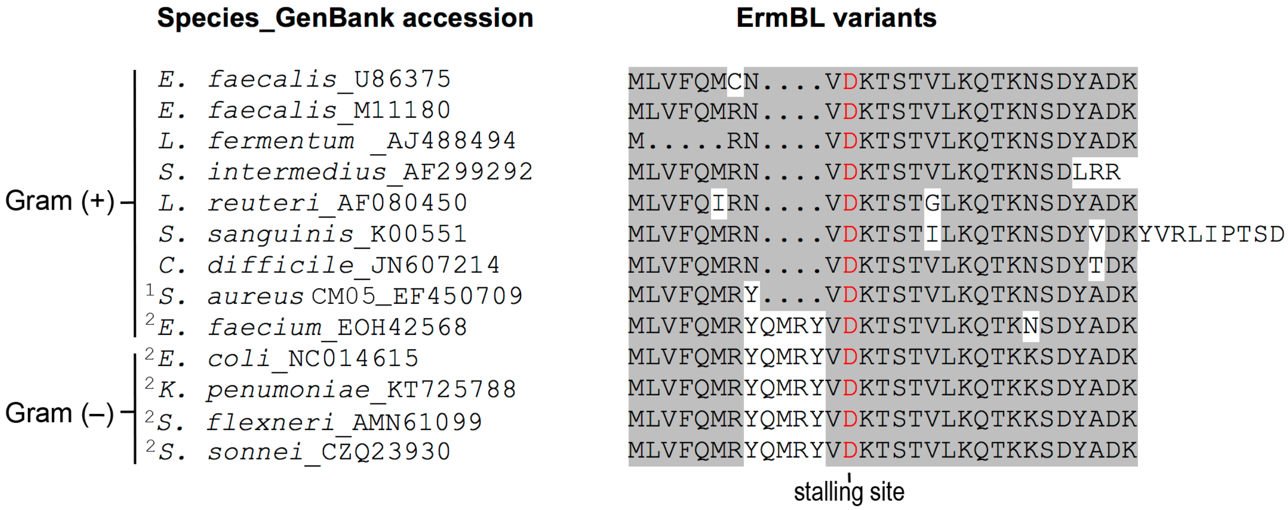

Figure S1. Multiple sequence alignment shows that ErmBL homologs are highly conserved. The ribosome-stalling site D10 is labeled in red. [1] $ErmBL_{EF}$ variant used in this study, this variant contains an Y8 in place of an N8. [2]Tandem duplication of “QMRY” renders high basal and inducible ErmB expression.

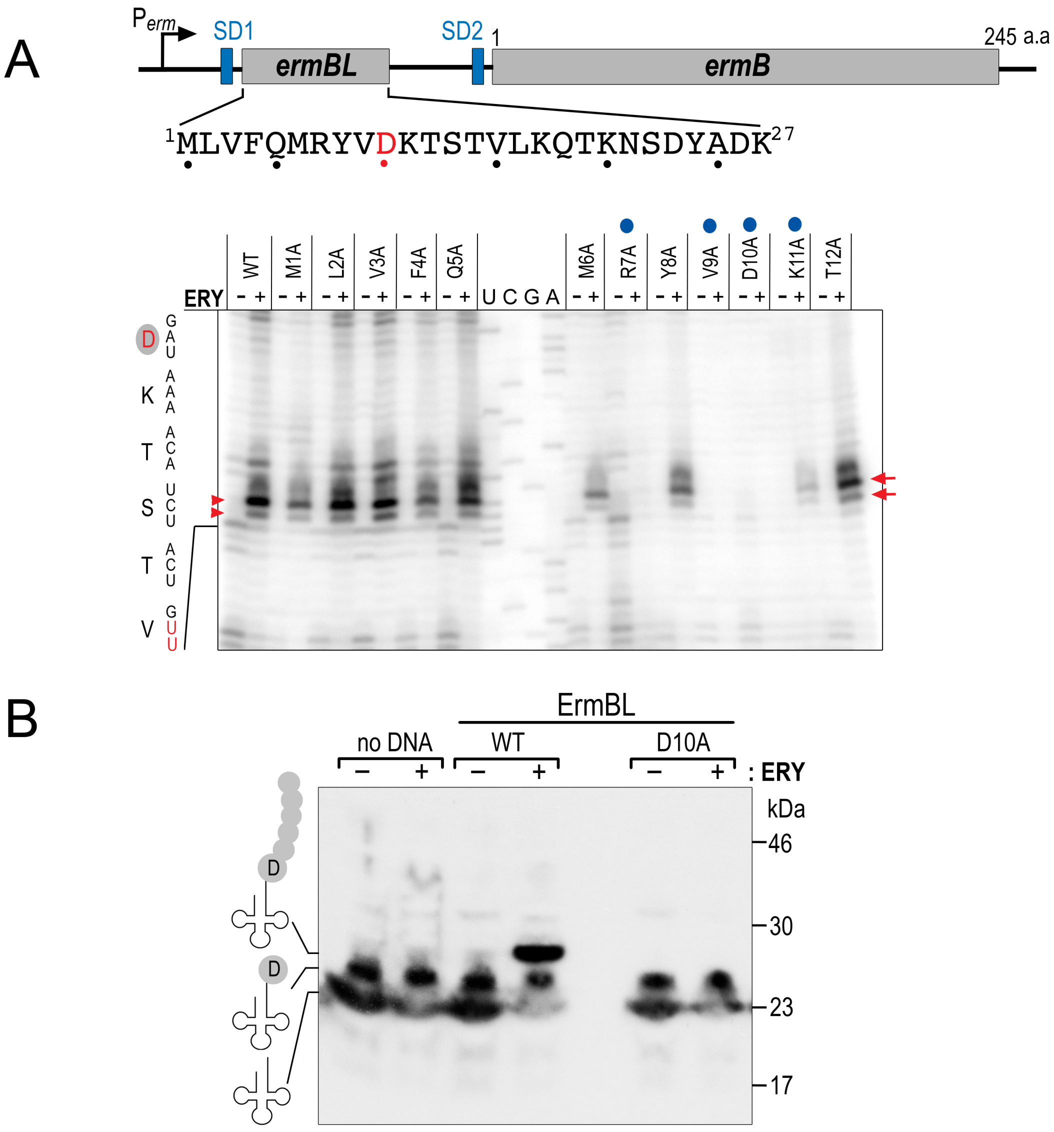

Figure S2. Toeprinting and Northern blot identify the stalling motif and stalling site of ErmBL$_{EF}$ leader peptide. (A) Alanine scanning mutants analyzed by toeprinting reveal the critical residues (R7, V9, D10 and K11, blue circles) necessary for full translation arrest. Red arrows mark the toeprint bands that confirm the P-site codon is an aspartate. **(B)** Northern blot shows that Asp10 is the last amino acid incorporated into the stalled nascent peptidyl-tRNA. The tRNA, aminoacyl-tRNA and peptidyl-tRNA are illustrated.

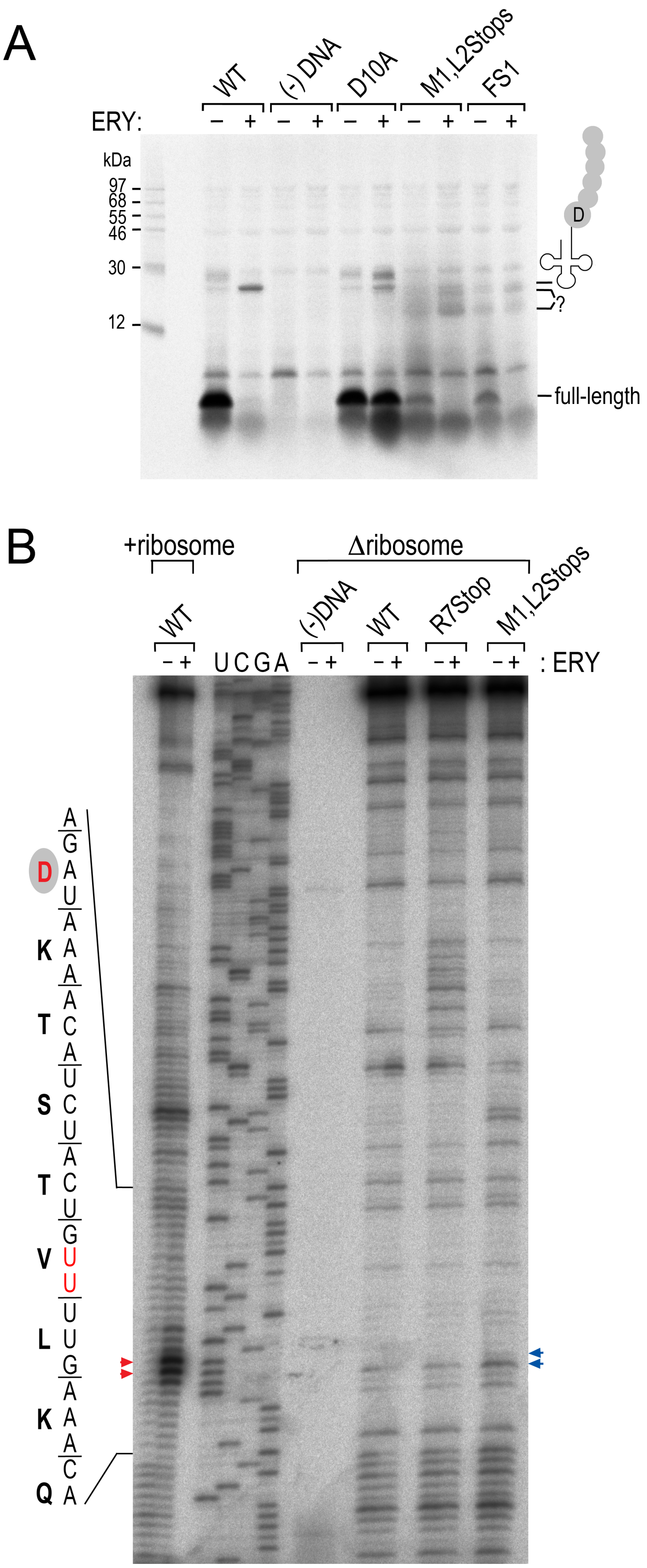
A
WT
(-) DNA
D10A
M1,L2Stops
FS1
ERY:
kDa
97
68
55
46
30
12
D
?
full-length
B
+ribosome
Δribosome
WT
U C G A
(-)DNA
WT
R7Stop
M1,L2Stops
: ERY
D
K
T
S
T
V
L
K
Q

Figure S3. Confirmation of the source of the background bands in the *ermBL$_{EF}$* toeprinting reactions. (A) *In vitro* translation of *ermBL$_{EF}$* derivatives with ^{35}S-methionine labeling. Stalled peptidyl-tRNA^{Asp} is stabilized in the presence of erythromycin (ERY), as indicated by a strong ~22 kDa band and the disappearance of the full-length ErmBL$_{EF}$. D10A mutant is impaired in ribosome stalling but not completely defective, thus the co-existence of the ~22 kDa peptidyl-tRNA^{Asp} and the full-length ErmBL$_{EF}$. Residual translational read-through is observed in the M1L2stop double mutant and the frameshift mutant FS1 in the absence of ERY. In the presence of ERY, additional unknown products of higher molecular weight (depicted in a question mark) are generated. **(B)** The omission of ribosome from the *in vitro* toeprinting reaction produces background bands (blue arrows). Red arrows mark the true ErmBL(D10) toeprints. Each untranslated *ermBL$_{EF}$* mutant mRNA appears to adopt distinct conformation relative to the WT, as shown from the patterns of the primer extension, however the structural variations are unaffected by ERY treatment.

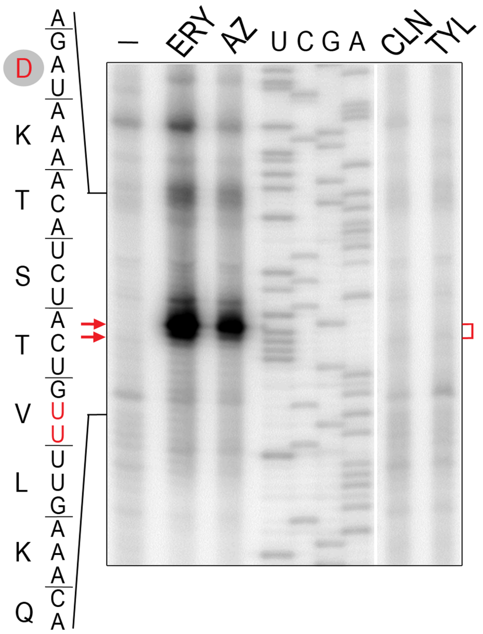

Figure S4. Erythromycin (ERY) and azithromycin (AZ) induce ErmBL$_{EF}$-dependent ribosome stalling *in vitro*, but clindamycin (CLN) and tylosin (TYL) do not. ERY, AZ and TYL belong to the 14-membered 15-membered, and 16-membered macrolide, respectively. CLN is a lincosamide. ERY and AZ were used at a final concentration of 50 μM and 25 μM, respectively. A range of CLN and TYL dosages (0-500 μM) was tested but only the data from 50 μM is shown. Red arrows mark the D10 toeprint signals.

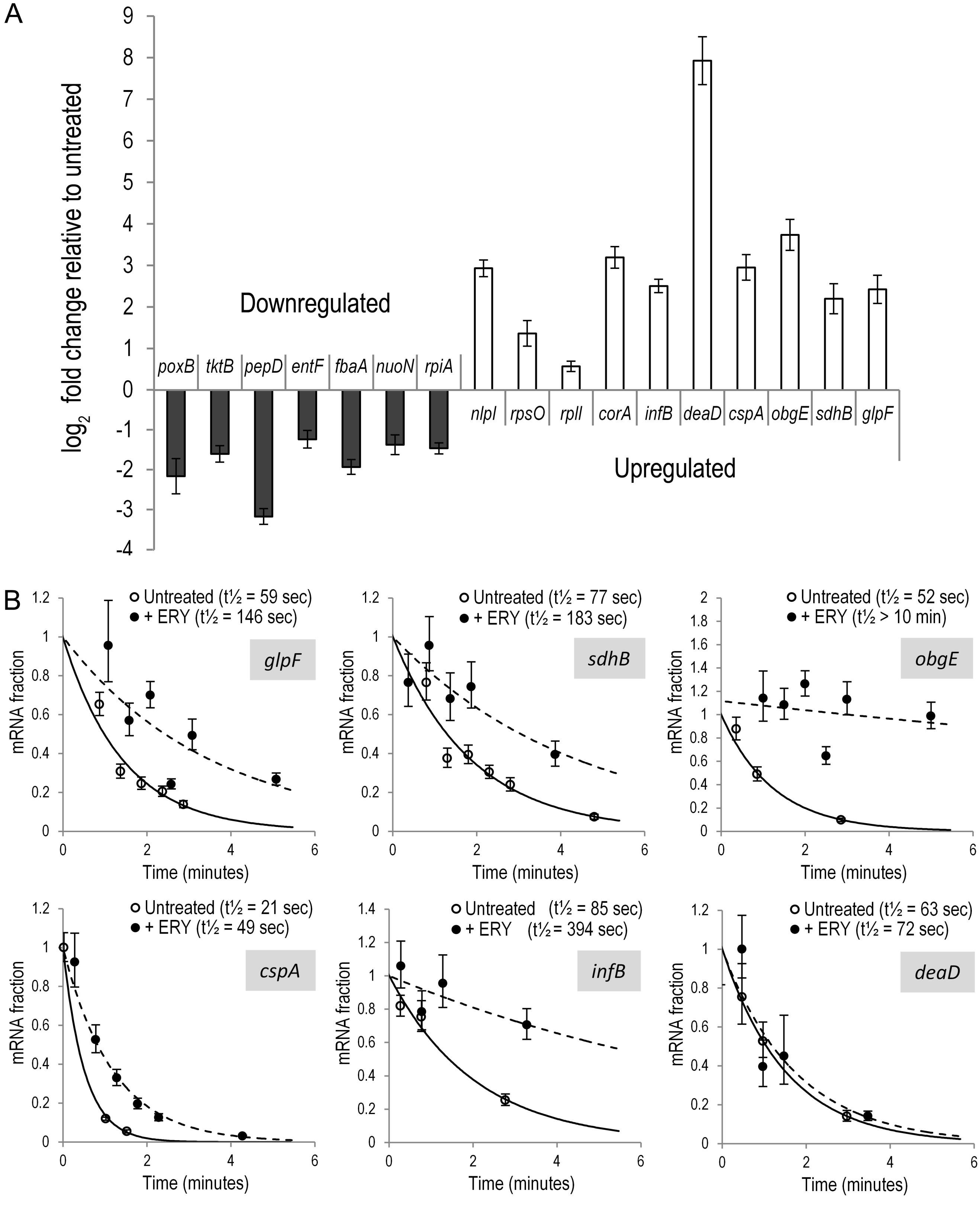
A
log2 fold change relative to untreated
Downregulated
Upregulated
poxB
tktB
pepD
entF
fbaA
nuoN
rpiA
nlpI
rpsO
rplI
corA
infB
deaD
cspA
obgE
sdhB
glpF
B
Untreated (t½ = 59 sec)
+ ERY (t½ = 146 sec)
glpF
Untreated (t½ = 77 sec)
+ ERY (t½ = 183 sec)
sdhB
Untreated (t½ = 52 sec)
+ ERY (t½ > 10 min)
obgE
Untreated (t½ = 21 sec)
+ ERY (t½ = 49 sec)
cspA
Untreated (t½ = 85 sec)
+ ERY (t½ = 394 sec)
infB
Untreated (t½ = 63 sec)
+ ERY (t½ = 72 sec)
deaD
mRNA fraction
Time (minutes)

Figure S5. Quantitative RT-PCR validates the RNA-seq results and reveals that the half-lives of a large fraction of antibiotic-upregulated genes are extended upon antibiotic treatment. (A) The trend and fold change of the ERY-affected genes are consistent with the RNA-seq data (see Dataset 1). **(B)** Rifampicin chase/qRT-PCR shows that except for *deaD*, all of the other 5 antibiotic-upregulated genes have increased mRNA half-lives by at least 2-fold. Error bars are standard deviation of mean from two independent biological replicates.

Table S1. Bacterial strains and plasmids used in this study.

Strains/plasmids	Relevant characteristics	Source
S. aureus		
CM05	Resistant to erythromycin, clindamycin, chloramphenicol, ciprofloxacin, dalfopristin, gentamicin, oxacillin, linezolid, and quinupristin.	BEI resources
E. coli		
DH5α	*supE44* Δ*lacU169* (Δ*80lacZ*Δ*M15*) *hsdR17 recA1EndA1 gyrA96 thi-1 relA1*	Clontech
XL1-Blue	*endA1 gyrA96*(nal^R) *thi-1 recA1 relA1 lac glnV44* F'[::*Tn10 proAB*$^+$ *lacI*^{q} Δ(*lacZ)*M15] *hsdR17*(r_K^- m_K^+)	Stratagene
MG1655	Wild-type K12	
NM580	MG1655 *lacI*-T1T2-*zeo*^{R}-pBRpLacO-kan-pBAD-*ccdB*. Mini-λ-Red::*tet*^{R}	Nadim Majdalani Ref. (1)
MNY55	NM580 *ermBL*(WT)-*ermB'-lacZ*	This study
KL29	NM580 *ermBL*(FS1)-*ermB'-lacZ*	This study
MNY57	NM580 *ermBL*(FS2)-*ermB'-lacZ*	This study
MNY59	NM580 *ermBL*(D10A)-*ermB'-lacZ*	This study
MNY61	NM580 *ermBL*(M1A)-*ermB'-lacZ*	This study
KL01	NM580 *ermBL*(M6A)-*ermB'-lacZ*	This study
SA03	NM580 *ermBL*(R7A)-*ermB'-lacZ*	This study
SA05	NM580 *ermBL*(Y8A)-*ermB'-lacZ*	This study
SA07	NM580 *ermBL*(V9A)-*ermB'-lacZ*	This study
KL03	NM580 *ermBL*(T12A)-*ermB'-lacZ*	This study
KL17	NM580 *ermBL*(M1Stop)-*ermB'-lacZ*	This study
KL18	NM580 *ermBL*(L2Stop)-*ermB'-lacZ*	This study
MNY64	NM580 *ermBL*(M1,L2Stops)-*ermB'-lacZ*	This study
KL19	NM580 *ermBL*(V3Stop)-*ermB'-lacZ*	This study
KL20	NM580 *ermBL*(F4Stop)-*ermB'-lacZ*	This study
KL21	NM580 *ermBL*(Q5Stop)-*ermB'-lacZ*	This study
KL22	NM580 *ermBL*(M6Stop)-*ermB'-lacZ*	This study
SA11	NM580 *ermBL*(R7Stop)-*ermB'-lacZ*	This study
KL23	NM580 *ermBL*(M1Stop)-*lacZ*	This study
KL24	NM580 *ermBL*(L2Stop)-*lacZ*	This study
KL25	NM580 *ermBL*(V3Stop)-*lacZ*	This study
KL26	NM580 *ermBL*(F4Stop)-*lacZ*	This study
KL27	NM580 *ermBL*(Q5Stop)-*lacZ*	This study
KL28	NM580 *ermBL*(M6Stop)-*lacZ*	This study
**Plasmids**		
pGEMT-Easy(pTA) derivatives	Ap^R; cloning vector	Promega
	pTA*ermBL*(WT)-*ermB*, Ap^R	This study
	pTA*ermBL*(L2Stop)-*ermB*, Ap^R	This study
	pTA*ermBL*(Q5Stop)-*ermB*, Ap^R	This study
	pTA*ermBL*(R7Stop)-*ermB*, Ap^R	This study
	pTA*ermBL*(D10A)-*ermB*, Ap^R	This study
	pTA*ermBL*-*ermB* (Y103A) , Ap^R	This study
pRB381	Ap^R, *lacZ* translational fusion plasmid	ATCC 77377
p381ermBL-B'	Ap^R, translational fusion P_{erm}-*ermBL-ermB'* on pRB381.	This study

Table S2. Primers used in this study

Primer	Sequence (5’-3’)	Application
p494f<br>p495r	GCG CGG TGA GCA CCG GAA CGG CAC TGG TCA ACT TGG CCA TAA GCT TAG AAG CAA ACT TAA<br>GGG TAA CGC CAG GGT TTT CCC AGT CAC GAC GTT GTA AAA CGA CGG GAT CCG TTA AAA AGT	homologous recombination to the *lacZ* locus of *E. coli* NM580 chromosome
p500f<br>p497r	TGATGAACAGGGTCACGTCGTCCCGGA<br>CGG TGC GGG CCT CTT CGC TA	colony PCR / sequencing primer with *E. coli* NM580 template
p719<br>p720<br>p721	AAG CCT GCG ACC AAT TGA TTA AAA GTC AAC TGC TC<br>CGA ACC CGC GAC CCC CTG CGT GAC AGG CAG GTA TTC<br>CCA AAC CAG TTG CGC TAC CAA GCT GCG	Lys-, Asp-, Pro-tRNA probes for Northern blots
p700r<br>p299r	GTC ACT GGT AGG AAT TAA TCT AAC G<br>GTT TGT AAT TTT AGT TAT CTG TTT A	Reverse primers for *ermBL* toeprinting
p195f<br><br>p262r	GAA ATT AAT ACG ACT CAC TAT AGG GAG ACC ACA ACG GTT TCC CTC TAG AAA TAA TTT TGT TTA ACT TTA AGA AGG AGA TAT ACC A<br>AGA ATA TTT TAT ATT TTT GTT CAT	Incorporating a T7 promoter to a toeprinting DNA template
p198f<br>p262r	TAA CTT TAA GAA GGA GAT ATA CCA ATG TTG GTA TTC CAA ATG CGT<br>AGA ATA TTT TAT ATT TTT GTT CAT	PCR a toeprinting template from plasmid or chromosome
p607f<br>p608f<br>p609f<br>p610f<br>p611f<br>p612f<br>p616f<br>p617f<br>p618f<br>p619f<br>p620f<br>p633f<br>p632f<br>p816f<br>p817f<br>p818f<br>p819f<br>p820f	TAA CTT TAA GAA GGA GAT ATA CCA GCG TTG GTA TTC CAA ATG CGT (M1A)<br>TAG TAA CTT TAA GAA GGA GAT ATA CCA TAG TTG GTA TTC CAA ATG CGT (M1Stop/TAG)<br>TAA CTT TAA GAA GGA GAT ATA CCA TAG TAA GTA TTC CAA ATG CGT (M1Stop,L2Stop)<br>TAA CTT TAA GAA GGA GAT ATA CCA ATG A TTG GTA TTC CAA ATG CGT (FS1)<br>TAA CTT TAA GAA GGA GAT ATA CCA ATG AA TTG GTA TTC CAA ATG CGT (FS2)<br>TAA CTT TAA GAA GGA GAT ATA CCA ATG TTG GTA TTC CAA GCG CGT (M6A)<br>TAA CTT TAA GAA GGA GAT ATA CCA ATG GCG GTA TTC CAA ATG CGT (L2A)<br>TAA CTT TAA GAA GGA GAT ATA CCA ATG TTG GCG TTC CAA ATG CGT (V3A)<br>TAA CTT TAA GAA GGA GAT ATA CCA ATG TTG GTA GCC CAA ATG CGT (F4A)<br>TAA CTT TAA GAA GGA GAT ATA CCA ATG TTG GTA TTC GCG ATG CGT (Q5A)<br>TAA CTT TAA GAA GGA GAT ATA CCA ATG TTG GTA TTC CAA ATG GCG (R7A)<br>TAA CTT TAA GAA GGA GAT ATA CCA ATG TAA GTA TTC CAA ATG CGT (L2Stop)<br>TAA CTT TAA GAA GGA GAT ATA CCA ATG TTG GTA TTC CAA ATG TAA TAT G (R7Stop)<br>TAA TAA CTT TAA GAA GGA GAT ATA CCA TAA TTG GTA TTC CAA ATG CGT (M1Stop/TAA)<br>TAA CTT TAA GAA GGA GAT ATA CCA ATG TTG TAA TTC CAA ATG CGT (V3Stop)<br>TAA CTT TAA GAA GGA GAT ATA CCA ATG TTG GTA TAA CAA ATG CGT (F4Stop)<br>TAA CTT TAA GAA GGA GAT ATA CCA ATG TTG GTA TTC TAA ATG CGT (Q5Stop)<br>TAA CTT TAA GAA GGA GAT ATA CCA ATG TTG GTA TTC CAA TAA CGT TAT G (M6Stop)	Alternate mutation-containing forward primers to create a toeprinting template
p827f<br>p828r	TGG GAA TAT TCC TGC CCA TTT AAG CAC A<br>TGT GCT TAA ATG GGC AGG AAT ATT CCC A	*ermB*(Y103A)
p289<br>p290	GCG TTA ACT CGG CGT TTC ATC<br>TCT CCG GCG CGT AAA AAT G	qPCR primers (*lacZ*)
p907f<br>p763r	GTC GAA CGG TAA CAG GAA GAA G<br>AAA GTT TTG AGA ATA TTT TAT AT	qPCR primers (5’-*ermB*)
p19f<br>p20r	TCG TCT GGA AAA AGC TGC AAC T<br>TAC GTC ATC TTC GGT GTA GCC C	qPCR primer (*gapA*)
p907f<br>p908r	GTC GAA CGG TAA CAG GAA GAA G<br>TGC GAC GTT ATG CGG TAT TAG	qPCR primers (16S rRNA)
p1010f<br>p1011r	CTC AGT ATG GAT GCG GTA CAA A<br>GGG TCG GTA GGG TTA TGT TTA AG	qPCR primers (*tktB*)
p1008f<br>p1009r	AGC ACA ACT TAG CGG AGA AC<br>AGG GAA TAT GAG CGG CAA TC	qPCR primers (*poxB)*
p1012f<br>p1013r	GAT TAA CAC CGA CTC CGA AGA A<br>GGA ACC GCT TCA CGA TCT AA	qPCR primers (*pepD*)
p1014f<br>p1015r	CTG ATA CAG GTG GCG GAT AAC<br>GGT AAT GGC CGG GAA ACT AA	qPCR primers (*entF*)
p1016f<br>p1017r	ACG GTG GTG CTT CCT TTA TC<br>ACA CCA TAA TGT TCA GCC ATC T	qPCR primers (*fbaA*)
p988f<br>p989r	GGT TTC GCT CTG GTT TGT TG<br>AAT ACC AGC CCG GTG TAA AG	qPCR primers (*nuoN*)
p990f<br>p991r	CAA TGA AAG GCC AGA TTG AAG G<br>GCT GTC GAC TTC GTT GAG AT	qPCR primers (*rpiA*)
P980f<br>P981r	CTC GAT GAG AAG CAG GCT AAA G<br>GCT AAT GTT GCC CAG GTA GAA	qPCR primers (*nlpI*)
p978f<br>p979r	TGA CTG CAC AGA TCA ACC AC<br>GCA GTT TAC GAC GCT GAG AA	qPCR primers (*rpsO*)

p976f<br>p977r	CTG GAA GCT AAA CTG GCT GAA<br>AGC TTT AGA CGC GAT GGT AAC	qPCR primers (*rplI*)
p974f<br>p975r	GCC TGC ATA TTC ACT CCT TCT<br>AGA GTA AAC AGA CGA CCA TCA C	qPCR primers (*corA*)
p972f<br>p973r	TGA AGA AGC ACG TCG TAT GG<br>GGC GAG CAT GTT GAG AAG TA	qPCR primers (*infB*)
p970f<br>p971r	CTC CAA TTC AGG CAG AGT GTA T<br>CAA GAT TCT GCA ACA GAG GTA AAG	qPCR primers (*deaD*)
p968f<br>p969r	AAG GCT TCG GCT TCA TCA C<br>CTG ACC TTC GTC CAG AGA TTT G	qPCR primers (*cspA*)
p966f<br>p967r	TGA AAC CAT GGG CGA TAT GA<br>GGA CGA TTT GAA ACG GGT ATT	qPCR primers (*obgE*)
p964f<br>p965r	CGC TAT AAC CCG GAT GTT GAT<br>CAG GCT GGG ATC TTT CTC TTT	qPCR primers (*sdhB*)
p962f<br>p963r	GGC TTT AGT TTA CGG GCT TTA C<br>AAG TGC CAG CCA GAT CAA	qPCR primers (*glpF*)
p1019r	CCC ACC TAT CCT ACA CAT CAA GGC TC	Reverse primer extension oligo (23S rRNA)
p805f<br>p806r	TGC GTA TGG TTA ACC CTA AAG TTA T<br>TTA TCT ACA TTC CCT TTA GTA ACG TG	Cloning of the full-length *ermBL-ermB* operon into pGEMT-Easy by TA cloning
p129f<br>p151r	TGA AGC TTA GAA GCA AAC TTA AGA GTG TG (HindIII)<br>TTT TGG ATC CGT TAA AAA GTT TTG AGA ATA TTT (BamHI)	Cloning of P_{erm}-*ermBL-ermB* into pRB381

SUPPLEMENTAL REFERENCES

1. **Battesti A, Majdalani N, Gottesman S.** 2015. Stress sigma factor RpoS degradation and translation are sensitive to the state of central metabolism. Proc Natl Acad Sci U S A **112:**5159-5164.
